# P003 Parenteral iron-induced hypophosphatemia osteomalacia (PITHO)/6H syndrome: a biopsy-proven case in intestinal failure

**DOI:** 10.1093/rap/rkag113.033

**Published:** 2026-11-03

**Authors:** Lih Yin Chong, Anupama Nandagudi

**Affiliations:** Basildon University Hospital, Basildon, United Kingdom; Basildon University Hospital, Basildon, United Kingdom

## Abstract

**Introduction:**

Hypophosphatemia osteomalacia is a recognizable complication of recurrent parenteral iron infusion, particularly ferric carboxymaltase (FCM). FCM induces biologically active fibroblast growth factor-23 (FGF23), increases urinary phosphate excretion and suppress 1,25-dyhydroxyvitamin D(1,25(OH)2 D) synthesis. This results in reduction of serum calcium and causes secondary hyperparathyroidism. These biochemical changes were recently summarised as 6H syndrome. We present a case of biopsy proven osteomalacia in a patient with long-term parenteral nutrition and repeated FCM infusions. This case highlights the diagnostic challenges, clinical consequences and importance of recognising parenteral iron triggered hypophosphatemia osteomalacia (PITHO).

**Case description:**

A 42-year-old man with congenital smooth muscle actin deficiency causing chronic intestinal pseudo-obstruction and intestinal failure was referred to a specialist metabolic bone clinic for evaluation of severe metabolic bone disease. He had been dependent on long-term total parenteral nutrition (TPN) and had received multiple intravenous iron infusions for recurrent iron deficiency anaemia, including ferric carboxymaltose in 2021, 2022, 2023 and 2025.

Initial investigation showed profound skeletal fragility. Dual-energy X-ray absorptiometry showed severe reductions in bone mineral density, with a lumbar spine T-score of -4.6, femoral neck T-score of -3.9 and total hip T-score of -3.4. Thoracic imaging showed multiple vertebral fractures, while CT imaging identified chronic compression fractures involving T12 and L4. Biochemical investigations showed persistent hypophosphataemia (0.79 mmol/L), hypocalcaemia, vitamin D insufficiency and elevated alkaline phosphatase (222 U/L), consistent with impaired bone mineralisation.

Given background of his intestinal failure and nutritional deficiencies, the aetiology of his metabolic bone disease was initially uncertain. A transiliac bone biopsy performed in December 2024 demonstrated extensive osteoid accumulation, abnormal mineralisation and excessive bone turnover, confirming hypophosphataemic osteomalacia. Histology also showed abundant haemosiderin deposition within the bone marrow.

Following multidisciplinary review by the Eastern Rare Bone Network, repeated ferric carboxymaltose exposure was identified as a major contributor through FGF23-mediated renal phosphate wasting. The diagnosis of parenteral iron-triggered hypophosphataemic osteomalacia (PITHO) was established. Recommendations included permanent avoidance of ferric carboxymaltose, consideration of alternative iron formulations, optimisation of phosphate, calcium and vitamin D replacement, monitoring of phosphate homeostasis and consideration of active vitamin D therapy. Ongoing management has focused on correction of mineral deficiencies and prevention of further skeletal deterioration.

**Discussion:**

PITHO is increasingly recognised but remains under-diagnosed, particularly in patients with complex gastrointestinal disease who receive recurrent intravenous iron. FCM is uniquely associated with marked increases in biologically active FGF23, triggering renal phosphate wasting, suppression of 1,25-dihydroxyvitamin D and secondary hyperparathyroidism. The resulting biochemical profile—recently termed the 6H syndrome—creates a sustained environment of impaired mineralisation that can progress to clinically significant osteomalacia.

This case illustrates several diagnostic challenges. First, symptoms of bone pain, fatigue and fractures are easily attributed to malnutrition, chronic illness or long-term steroid-free fragility, delaying recognition of a reversible metabolic cause. Second, hypophosphataemia is frequently overlooked in routine blood panels, and even when detected, its significance may be underestimated. In patients with intestinal failure, multiple competing factors—malabsorption, vitamin D deficiency, chronic inflammation and TPN composition—can obscure the contribution of FGF23-mediated phosphate wasting.

The bone biopsy demonstrating classical features of osteomalacia including excess osteoid and defective mineralisation. Haemosiderin deposition provided an important clue to repeated iron exposure. Multidisciplinary review enabled synthesis of the clinical, biochemical and histological findings, leading to the correct diagnosis of PITHO.

Clinically, the consequences were profound: severe reductions in bone mineral density, multiple vertebral fractures and ongoing skeletal fragility. Importantly, the patient had received FCM over several years, highlighting that cumulative exposure increases risk, particularly in individuals with coexisting vitamin D insufficiency or limited dietary phosphate intake.

**Key learning points:**

• Acquired causes of hypophosphataemia should be considered in patients presenting with unexplained bone pain, fragility fractures, or severe reductions in bone mineral density.

• Although osteoporosis is frequently considered when low bone mineral density and fractures are identified, osteomalacia may present similarly and requires different management. Failure to recognise the distinction may lead to inappropriate treatment and progression of disease.

• A key learning point is that ferric carboxymaltose is increasingly recognised as the intravenous iron formulation most strongly associated with FGF23-mediated phosphate wasting. Clinicians prescribing recurrent iron infusions should be aware of this adverse effect and consider routine phosphate monitoring in high-risk patients.

• This case also demonstrates the value of bone biopsy in complex metabolic bone disease. Histological confirmation provided diagnostic certainty when multiple potential causes were present and directly influenced subsequent management decisions. Although invasive, bone histomorphometry remains the gold standard for diagnosing osteomalacia in selected cases.

• The case has highlighted the importance of multidisciplinary management involving metabolic bone specialists, gastroenterologists, nutrition teams and primary care clinicians. Close collaboration was necessary to interpret biochemical abnormalities, optimise nutritional support and minimise further exposure to causative agents.

